# Supplementary material for: Chromothripsis during telomere crisis is independent of NHEJ, and consistent with a replicative origin
Source: Genome Res. 2019 May;29(5):737–49. doi: 10.1101/gr.240705.118 (PMC6499312; doi:10.1101/gr.240705.118)
Supplement: Supplemental Material [file supp_gr.240705.118_Supplemental_file_1.zip › contigs/annotated_contigs/DB51/contig.2.DB51_length_673_mean_cov_10.0995542348.docx]

**DB51_length_673_mean_cov_10.0995542348**

```
TGGAACTGTAAGTCCATTAAACTTCTTTCTTTTGTAAATTTCCCAGTCTCAGGTTTGTCTTTATCAGCTGCATGAAAAAGGAATAATAC
 >chr8:88798038-88798371 - E=1e-188
AGTAAATCAGTACCAGGAGTGGGGTGTTACTGAAAAGATACCCAAAAATGTGTAAGCAACTTTGGAACTGGGTAGTAGGCAGAGGTTGG

AACAGTTTGGGGGGCTCAGAAGACAGGAAAATGTGGGAAAGTTTAGAACTTCCAGAGACTTGTTGAATGGTTTTGACAAAAATGCTGAT

AATGATATGAACAATAAGGTTCAAGCTGAGGTGGTCTCAGATGGAGATGAGAAACTTGTTTGGA|AC|ATGGAGTCAGAGGGGATCATT
                                                                 >chr8:88796717-8879705
TTGGAGCTTTAAGATTTGACTGCATTGCTGAATTTTGGACTTGCATGAGGCCTGTAGCCCCTTTGTTTTGGCCAATTTCTCTGATTTAG
9 - E=5e-194
AATGGCTGTATTTACCCCAATGTCTGTACCCCCATTGTATCTAGGAAGTAACTAACTCACTTTTGATTTTACAGGCTCATAGGCTGAAG

GATCTTGCTTTGTCTTGGATGAGACTTTGGACTGTGGAATTTTGAGTTAATGCTGTAATGAGTTAAGACTTTGGGAGACTGTTGGGAAG

ACATTTGGTATGAAATGTGAGGACATGAGATTTGCAAGGGGCCGGGGTGGAA
```
